# Automated microbeam observation environment for biological analysis—Custom portable environmental control applied to a vertical microbeam system

**DOI:** 10.1016/j.snb.2016.08.076

**Published:** 2016-08-16

**Authors:** Matthew J. England, Alan W. Bigelow, Michael J. Merchant, Eirini Velliou, David Welch, David J. Brenner, Karen J. Kirkby

**Affiliations:** a Ion Beam Centre, University of Surrey, Guildford, Surrey GU2 7XH, United Kingdom; b Center for Radiological Research, Columbia University, 630 West 168th Street, New York, NY, USA; c Christie NHS Foundation Trust, Manchester, United Kingdom; d Bioprocess and Biochemical Engineering Group (BioProChem), Department of Chemical and Process Engineering, University of Surrey, United Kingdom; e University of Manchester, Institute of Cancer Sciences—Manchester Academic Health Science Centre, Manchester, United Kingdom

**Keywords:** Microbeam, Time-lapse microscopy, Environmental control, Arduino, 3D printing

## Abstract

Vertical Microbeams (VMB) are used to irradiate individual cells with low MeV energy ions. The irradiation of cells using VMBs requires cells to be removed from an incubator; this can cause physiological changes to cells because of the lower CO_2_ concentration, temperature and relative humidity outside of the incubator. Consequently, for experiments where cells require irradiation and observation for extended time periods, it is important to provide a controlled environment. The highly customised nature of the microscopes used on VMB systems means that there are no commercially available environmentally controlled microscope systems for VMB systems. The Automated Microbeam Observation Environment for Biological Analysis (AMOEBA) is a highly flexible modular environmental control system used to create incubator conditions on the end of a VMB. The AMOEBA takes advantage of the recent “maker” movement to create an open source control system that can be easily configured by the user to fit their control needs even beyond VMB applications. When applied to the task of controlling cell medium temperature, CO_2_ concentration and relative humidity on VMBs it creates a stable environment that allows cells to multiply on the end of a VMB over a period of 36 h, providing a low-cost (costing less than $2700 to build), customisable alternative to commercial time-lapse microscopy systems. AMOEBA adds the potential of VMBs to explore the long-term effects of radiation on single cells opening up new research areas for VMBs.

## 1. Introduction

The environment for *in vitro* cell culture is typically an incubator at 37 °C, 5% CO_2_ and a relative humidity of 97–100% [1–3]. Observation of cells with a microscope requires them to be removed from an incubator. This change in environmental conditions experienced by the cells during an experiment is sub-optimal, especially where long-term experiments are desired, to observe factors such as cell motility, mutagenesis or the effects of radiation over several cell cycles. When cells are removed from the incubator lower CO_2_ levels in laboratory conditions lead to a rise in pH, lower relative humidity leads to a higher rate of evaporation of cell medium, and the change in temperature disrupts cellular metabolism [4]. Noticeable changes in cell health can occur as soon as five minutes after removal from the incubator and can eventually lead to cell death [1,3]. To combat the effects of removing the cells from the incubator, specialised time-lapse microscopes can be purchased that incorporate dedicated environmental control systems. Off-the-shelf time-lapse systems usually fall into two main categories: they are either an incubator that contains a microscope, an example of this type of system is the Nikon BioStation IM-Q (Nikon, Minato, Tokyo, Japan), or they are microscopes with environmental chambers, such as the Cell Observer system by Zeiss (Zeiss, Oberkochen, Stuttgart, Germany) or the Leica DMi8 Live Cell Imaging System (Leica, Wetzlar, Giessen, Germany). Costing up to $150,000, these high-cost systems have driven scientists to design their own time-lapse system on a lower budget, an example is the LOCOMOTIS (Low-Cost Motility Tracking System) produced by Lynch et al. [5]. The system, built for only € 200 (£161), was capable of viewing up to three cell dishes simultaneously with built-in temperature control. Walzik et al. created a webcam-based system intended for use in schools that cost less than € 1250; the system was able to observe an entire cell dish for 48 h with full time-lapse capabilities with both dark and bright field imaging scanning across the dish with an Arduino controlled XY stage [6]. It was also able to control relative humidity, CO_2_ and temperature to recreate incubator-like conditions.

Vertical Microbeams(VMBs) such as at Columbia University’s Radiological Research Accelerator Facility (RARAF) or at Surrey University’s Ion Beam Centre comprise the disciplines of ion-beam physics, radiation biology and microscopy. This makes them unique tools that allow for the simultaneous observation and irradiation of cells using particle radiation [7,8]. Experiments at these facilities occur outside of an incubator thus, these systems face the same challenges as conventional microscopy systems, with the added complication of coupling the microscope to the end of a vertical ion-beam line. Since VMBs require highly modified or their own custom designed microscope systems, it is therefore not feasible to use an off-the-shelf time-lapse microscope system on a VMB [8–10].

The Automated Microbeam Observation Environment for Biological Analysis (AMOEBA) was developed to integrate environmental control into VMBs. The AMOEBA is a modular environmental control system which taps into the vast resources of the “maker” community reducing development costs and allowing easy reproduction of parts, ultimately creating a system that can be reproduced in other laboratories [11]. By taking advantage of versatile programming environments like the Python Programming language (Python 2.7, https://www.python.org/) and the Arduino environment (Arduino, https://www.arduino.cc/), computer-automated manufacturing techniques, for example, 3D printing, and widely available sensors to allow for quick development times the AMOEBA system provides a low cost homemade alternative to the high cost commercial systems. AMOEBA’s open source nature, relatively low cost (costing less than $2700) and flexibility also make AMOEBA a useful technology for the new Do-It-Yourself Biology (DIYBio) movement allowing experimental capabilities that cost $100,000 s to be carried out on equipment of that costs a fraction of that [12,13]. The AMOEBA system provides a novel environmental control system that unlocks the potential of VMBs, allowing for future analysis of the long-term effects of radiation on single cells without having to disturb the cells from the end station.

## 2. Methods and materials

### 2.1. The AMOEBA system

**Fig. 1:**
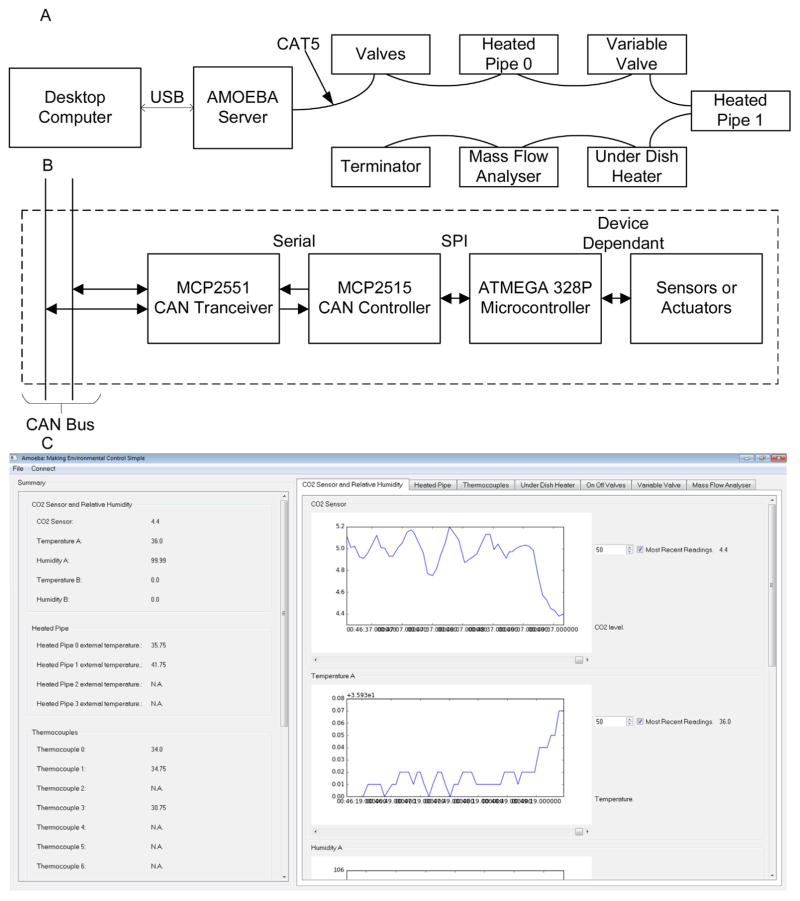
(A) A schematic of the entire AMOEBA system, showing the bus consisting of a group of modules daisy-chained together with CAT5 cable that carries a common CAN bus and power supply. (B) A block diagram showing the structure of an AMOEBA module. Each module has a sensor or actuator attached to it; how the microcontroller interfaces with these vary between modules. (C) A screen shot of the AMOEBA desktop client showing CO_2_ and air temperature data from an AMOEBA experiment.

The AMOEBA was designed with the flexibility to be applied to other applications without having to write any additional code. This meant that different sensors and actuators could be configured independently depending on the task at hand. This flexibility was achieved by designing the system around a single Controller Area Network (CAN) bus to which all the devices are connected, a schematic is shown in Fig. 1A. To run an experiment, the modules on the bus are configured by the server module. During the experiment, each device communicates with the other devices on the bus using a common interface protocol. The single bus design allows new modules to be added to the system with ease and also allows the user to reconfigure the system in a matter of minutes.

The AMOEBA has two main parts, the desktop software and a collection of different AMOEBA modules that connect together using CAT 5 cable. The server module connects the AMOEBA system to a computer using a USB (Universal Serial Bus) cable and each of the other modules has a different sensor or actuator attached.

### 2.2. Design and constructing of an AMOEBA module

The structure of an AMOEBA module can be seen in Fig. 1B. Each AMOEBA module contains a single ATMEGA328P microcontroller (Atmel, San Jose, CA) that is programmed using the Arduino environment. The microcontroller interfaces with the CAN bus using a Microchip MCP 2515 CAN controller (Microchip Technology, Chandler, AZ) and an MCP 2551 CAN transceiver (Microchip Technology, Chandler, AZ). In terms of the Open Systems Interconnection(OSI) model the MCP2551 transceiver and MCP2515 controller handle all the data link level requirements of the AMOEBA system while the ATMEGA328P handles the higher communication layers [14].

The single ATMEGA 328P microcontroller used in each AMOEBA module was chosen as the standard AMOEBA microcontroller for many reasons. First, the microcontroller is capable of controlling devices that operate using many standard bus protocols including I2C, SPI (Serial Peripheral Interface), and serial communication. It is also capable of reading analogue signals and outputting pulse width modulated signals that can be easily converted to an analogue output. These microcontrollers are compatible with the Arduino programming environment, which is widely used by the “maker” community so numerous resources exist to interface with off-the-shelf sensors, significantly reducing development time. The Arduino software is designed to be used by those who do not have a background in electronics, so it hides a lot of the intricacies of developing embedded code from the user. This simplified setup will allow 3rd party users who wish to use the AMOEBA to more effectively implement their own sensors. The Arduino programming environment is also useful because it works across a range of ATMEGA microcontrollers; this means that the code designed for the ATEMGA 328P can easily be used on a more powerful ATMEGA microcontroller if a module requires it.

A CAT5 cable is used for communication between the modules and to supply power. Of the eight wires available in a CAT5 cable three are used for ground, two are used for power, two are used for the CAN bus, and one is spare. If any module requires more than 0.5A then an external power supply can be connected to the device.

### 2.3. Types of AMOEBA modules

There are four different types of AMOEBA modules that are connected to the bus: sensor modules, actuator modules, server modules and terminator modules. Sensor modules are modules that monitor system parameters, such as temperature. Actuator modules are modules that control a parameter, for example, a variable valve or a heater. The server module connects the AMOEBA system to a computer running the AMOEBA desktop client by a USB interface; it contains the main power supply and sets up the bus at the start of the experiment and can interface with up to 254 other modules. Finally, there is a sole terminator module used to terminate the AMOEBA bus. Each module on the AMOEBA system communicates using a standard protocol designed for the AMOEBA system, this means that any actuator module can process the data gathered by any sensor module. With this modular approach, the AMOEBA system can be quickly configured to suit any application.

### 2.4. Control system on a bus

The AMOEBA system uses a CAN 2.0 bus that links all the modules together. The packets used are structured so that each module can communicate with any other module. This is achieved by using the first byte in each packet to designate the packet type. Each module also has its own unique address and a common shared address that can be used to send commands to other modules. The majority of the AMOEBA modules are either sensor or actuator modules, these are used to control or monitor various parameters. At the start of an experiment, each controller module on the bus is configured by the server module to monitor a certain sensor on the bus and the set point of that sensor. Once a start command is received the actuator module repeatedly polls the sensor module and adjusts its output accordingly. This continues until the actuator receives a stop command. We configured the AMOEBA system to work in a polled mode, it could easily be modified to operate in a triggered mode although this would not be required for environmental control since environmental control on cell culture does not require a fast response time.

### 2.5. AMOEBA desktop client

A computer running the AMOEBA desktop client connects to the server module by a USB cable. The AMOEBA software records the data gathered by the sensors and sets up the bus to control the environment. Once gathered, the data can be saved either into a specific AMOEBA XML file or to a character separated file that can be read using a customised script or any commercial spreadsheet software. For more complex experiments, the AMOEBA desktop client is also capable of running customised Python scripts specific to the experiment. Fig. 1C shows a screen shot from the AMOEBA software client which was written in Python 2.7 with the graphical user interface created using the PySide library (PySide, Qt, Santa Clara, CA) and Matplotlib [15] used to create the graphs.

### 2.6. Applying the AMOEBA system to the task of environmental control

Recreating incubator conditions on the end of a microbeam requires the control of 3 parameters: relative humidity, CO_2_ concentration and cell medium temperature. This was achieved by creating a controlled volume above the cells in which relative humidity and CO_2_ are controlled and by heating the cell dish to regulate cell medium temperature.

**Fig. 2:**
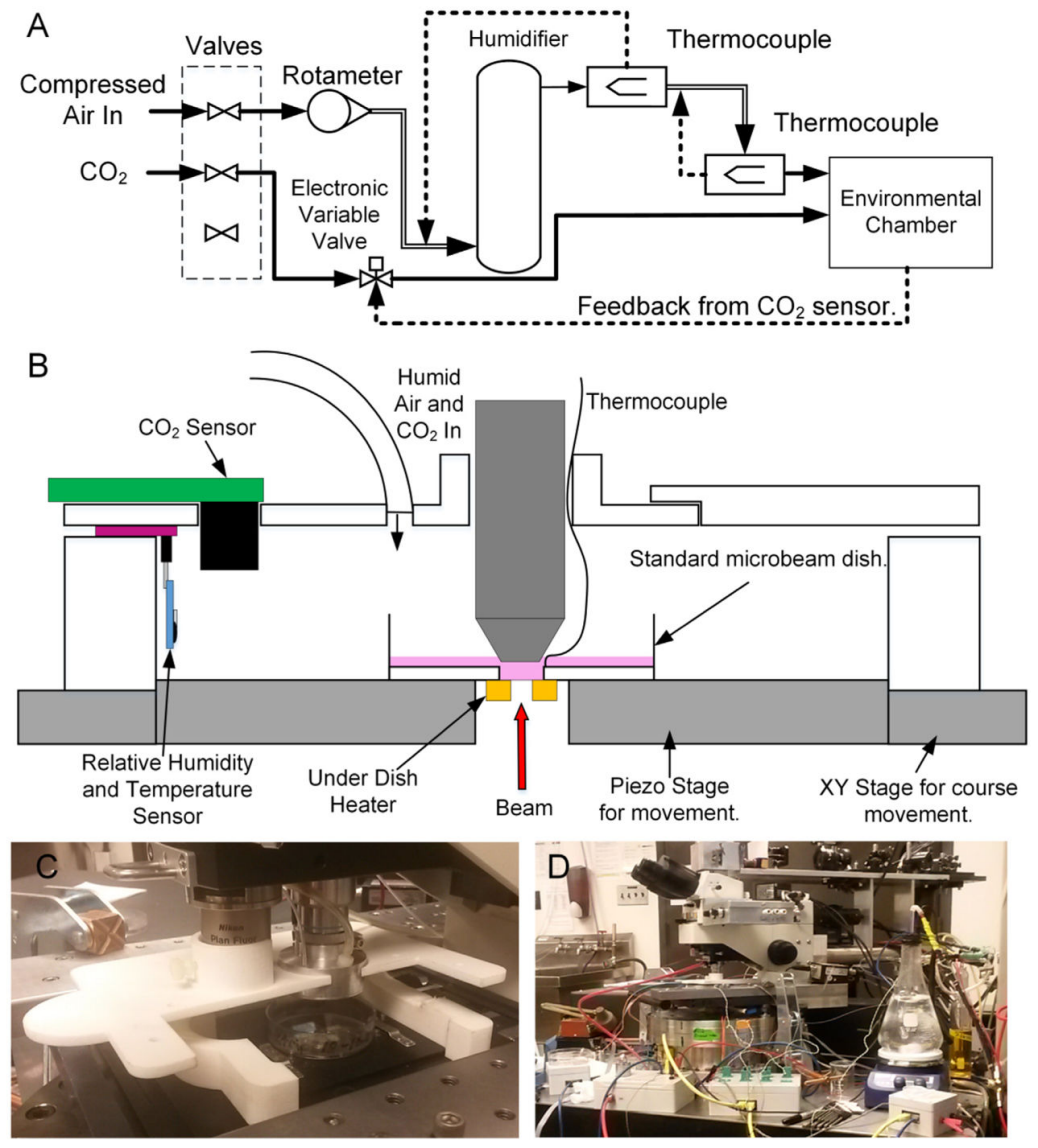
(A) A schematic of the gas flow control system of the AMOEBA. Solid lines signify a pipe, the double lines are heated pipes. The dotted lines show feedback in the system, used to control the gas at each stage of the system. When an experiment has started the valves on the left-hand side are opened to allow gas to enter the system. (B) A side view schematic of the environmental chamber used in the AMOEBA system. The chamber is designed such that the right-hand side can be removed to aid the loading of cell dishes. (C) A photograph of the environmental chamber on the end of one of RARAF’s Microbeam with the side removed to allow the microbeam cell dish to be changed. (D) The AMOEBA system on the end of a RARAF microbeam.

The processes used to create the humid, CO_2_ rich atmosphere can be seen in Fig. 2A. The gases enter the system (at room temperature) from gas cylinders, the gas flow is turned on when the experiment starts using two solenoid valves in the solenoid valve module. The compressed air that enters the system is heated in a section of heated pipe before being bubbled through a humidifier; this creates air with a relative humidity of 100%. Upon leaving the humidifier, the air is transported to the environmental shroud by another section of heated pipe to minimise air-cooling and condensation. K-type thermocouples are used at all stages to monitor temperature. The concentration of CO_2_ within the shroud is read by a CO_2_ sensor (Gas Sensing Solutions SprintIR 100% (Gas Sensing Solutions, Westfield North Courtyard, Cumbernauld, UK)) and maintained (e.g. at 5%) by the variable valve module that controls the flow of CO_2_ from a gas cylinder. The humid air and CO_2_ mix within the environmental shroud to create a humid CO_2_ rich atmosphere.

### 2.7. AMOEBA environmental shroud for the RARAF AMOEBA system

An AMOEBA environmental shroud (Fig. 2) is used to create a controlled but not sealed volume above the cell dish and to hold sensors in place during experiments. The shroud was designed using FreeCAD (FreeCAD: An open-source parametric 3D CAD modeller) and constructed using 3D printing. The design can be modified and reprinted within 6 h at a cost of less than $10 by users who possess their own extrusion 3D printer using either extruded ABS or PLA, or a professional service could produce the design for a cost of approximately $100. In the AMOEBA system described in this paper, the shroud was printed using the Shapeways 3D printing service using their Strong and Flexible plastic (Shapeways, NY) using a powder bed method. Although the shroud is not biocompatible this is not an issue because it is never in contact with the cells. The chamber is also not sealed but the air flow out is greater than the airflow into the chamber and also the amount of CO_2_ required to maintain 5% atmosphere is very low (0.05 l per hour); but if desired it would be possible to design and build a sealed chamber if desired.

This work uses a shroud designed around the RARAF microbeam endstation to validate the AMOEBA [7,10]. The RARAF environmental shroud allows for dual objectives as well as a removable side for easy removal and insertion of cell dishes. The external dimensions of the shroud are 12.3 cm × 12.3 cm × 2.3 cm, and it controls a volume of 167.4 cm^3^. To construct it uses a total volume of 157 cm^3^ of material to print with 100% infill. It is compatible with cell dishes with a diameter below 86 mm and could be easily be redesigned to cater for other plating systems if necessary without having to change any of the electronics.

### 2.8. AMOEBA modules on the RARAF AMOEBA system

Since a variety of factors need to be controlled using the RARAF AMOEBA, it uses a variety of self-contained independent modules each serving their own purposes. The sensor modules are thermocouples, relative humidity and CO_2_ monitoring, and mass flow analyser. The actuator modules are a variable valve, under dish heater, heated pipes and a solenoid valve module. There is also a sole server and terminator module. Collectively these modules cost $2380 to construct including the cost of the sensors.

The **thermocouple module** is a bank of eight thermocouples, where each channel contains its own MAX31855KASA+ (Maxim Integrated, CA) thermocouple preamplifier, which communicates with the microcontroller using an SPI bus with a maximum sensor refresh time of 100 ms. Although eight thermocouples are available the RARAF AMOEBA configuration uses four thermocouples: two are used to monitor the temperature of the compressed air, one is used to measure cell media temperature, and the fourth is used to measure the temperature of the water in the humidifier. The two thermocouples used to measure the temperature of the compressed air were adapted so that they can fit inside the airline by sealing it inside a Wye Push to Connect gas fitting using Permapoxy 5 min General Epoxy Resin (Permatex, CT). The Thermocouples module cost $120 to build and the thermocouples cost $90. It has a mass of 0.477 kg.

The **relative humidity and CO**_2_ **monitoring** is a single module with two HYT 271 relative humidity and temperature probes (Innovative Sensor Technology IST AG, Ebnat-Kappel, Switzerland) and a Gas Sensing Solutions SprintIR 100% CO_2_ sensor. The HYT 271 communicate with the module’s microcontroller via an I2C bus with a 1 s sampling time. The CO_2_ sensor communicates with the AMOEBA microcontroller using UART (Universal Asynchronous Receiver/Transmitter) serial communication with a 50 ms sampling time. During most AMOEBA experiments only one relative humidity and the ambient temperature sensor is used since the relative humidity and ambient temperature is only measured inside of the AMOEBA chamber. The module cost $45 to build, each of the Relative Humidity and Temperature sensors costs $36 each and the CO_2_ sensor costs $155. The module has a mass of 0.266 kg.

The **variable valve module** is used to control the flow rate of the CO_2_ gas. The module uses an FSV12 (Omega, Stamford, CT) electronically controlled proportional valve that allows a maximum flow rate of 13LPM (216.7 cm^3^ s^−1^) of air. The behaviour of the valve is controlled using a Proportional-Integral-Derivative (PID) control algorithm on the module’s microcontroller that can be tuned by the user depending on the application [16]. The variable valve cost $560 and the control electronics and housing cost $40 to manufacture. The module has a mass of 1.012 kg.

In microbeam experiments, the dish containing cells is often in close proximity to a metallic section of beam pipe that can serve as a major heat sink. The **under dish heater module** is a MOSFET switch that controls the current going through an under dish heater, created by wrapping nichrome wire around the brass cap protecting the microbeam exit window. The under dish heater module cost $43 to construct. The mass of the module is 0.218 kg.

The **heated pipe module** heats air to create the humid environment necessary for the cells. Three 1 m sections of heated pipe were created by wrapping nichrome wire around 0.25-inch outer diameter PTFE (Polytetrafluoroethylene) pipe. The pipe is heated by passing an electrically controlled current through the wire that surrounds the pipe. Each section of heated pipe is attached to a heated pipe control board, which contains a MOSFET switch to control the current through the Nifethal 70 wire by pulse width modulation and an onboard MAX31855KASA+ (Maxim Integrated, San Jose, CA) thermocouple preamplifier to monitor the external temperature of the pipe. Each section of heated pipe cost $40 to build. Each heated pipe module has a mass of 160 g.

The **solenoid valve module** for gas control consists of three solenoid valves controlled using the digital output pins on the microcontroller and they are driven using MOSFET switches. The valves are used to turn the CO_2_ and compressed airflow on at the start of an experiment and to turn them off at the end of the experiment. The three valves cost $200 and the electronics to control them cost $50. The mass of the module is 1.239 kg.

The **server module** is used to interface between the AMOEBA software and the AMOEBA modules. The server module contains an Arduino Uno that was connected to additional CAN circuitry so it could interface with the AMOEBA modules. It also contained a 15 V 10A power supply that provides the power for all the AMOEBA modules. The cost of the server module was $125. The mass of the server module is 1.432 kg.

CAN buses need to be terminated to achieve their best operation; as a result, the AMOEBA system has a **terminator module** that contains a resistor that is used to terminate the bus. The terminator modules cost $15 to construct. The terminator has a mass of 0.1 kg.

### 2.9. Additional AMOEBA modules

The AMOEBA system is also capable of interfacing with pre-existing lab equipment through the use of communication protocols such as GPIB, RS-232, USB or Ethernet. An example of this is the RS-232 AMOEBA module. It was created to interface with the JENCO 6230N handheld metre that connects to a range of ion sensors that can be used to measure factors such as dissolved oxygen or pH. This can be achieved due to the flexibility of the ATMEGA328P microprocessors used by each module and a large amount of freely available code already available online.

A **mass flow analyser module** is available to measure gas flow although it is not often used because the flow rates it can measure is too low. A Dwyer Instruments GFM-1108 Gas Mass Flow Metre (Dwyer, Michigan City, IN) is used and it outputs a 0–5 V signal that is read by the module’s microcontroller and converted to a flow rate. The GFM-1108 is capable of measuring airflow up to 2 l per Minute (LPM) (33.3 cm^3^ s^−1^). It is also capable of measuring other gases using the supplied calibration factor. The mass flow analyser cost $785 and the control electronics cost $40 to produce. The module had a mass of 930 g.

### 2.10. Testing the AMOEBA system

The application of the AMOEBA system for environmental control on the RARAF VMBs was tested in two steps. The first step was to test the ability of the AMOEBA system to create and sustain an incubator like (37 °C ±2 °C, 5% ± 0.5% CO_2_ and 99% ± 1% relative humidity) environment. To test the stability of the AMOEBA system, an AMOEBA experiment was set up using a cell dish containing 2 ml of cell media and run for 6 h; the dish was removed from the environmental shroud every 2 h to measure the mass of the cell dish. The second step was to test its ability to sustain cells within the device. To test this, cells were observed in the AMOEBA system for 36 h, with an image being taken every 10 min.

### 2.11. Preparation of cells for AMOEBA experiments

The protocol for growing the cells used to test the AMOEBA system was kept consistent with established protocols and is summarised briefly here [17].

HeLa (ATCC, VA) cells were first grown in DMEM (DMEM, Thermo Fisher Scientific) supplemented with 10% Foetal Bovine Serum (FBS, Thermo Fisher Scientific) and 1% of Pen/Strep (Pen-Strep, Thermo Fisher Scientific) using T75 culture flasks in a 5% CO_2_ atmosphere at 37 °C. Between 100 and 300 cells were plated onto a standard microbeam dish following standard protocol [17]. The microbeam dishes used were 60 mm Petri-dishes (Petri Dish 60 × 15 mm, Falcon) with a 6.35 mm diameter hole drilled in them and a 3.9 μm thick piece of polypropylene (0907P, Steinerfilm) stretched over this hole held in place with a thin layer of Master Bond EP30MED epoxy (Master Bond, NJ). The dishes were treated with Cell-Tak (3.5 μg cm^−2^) (DB Biosciences, CA) on the polypropylene sheet and cells were added on top of this.

The dish wells coated with Cell-Tak were coated by putting 10 μl of 100 μg ml^−1^ Cell-Tak solution and 10 μl of 0.2 M sodium bicarbonate solution (Sodium Bicarbonate, Gibco) into to the well. The dish was then placed in a Series 8000 Direct-Heat CO_2_ Incubator (Thermo Fisher Scientific) at 37 °C and 5% CO_2_ for 30 min. Once removed from the incubator the dish was rinsed 3 times with 5 ml of sterile phosphate buffered saline solution. After this, the dishes can be stored for up to two weeks in a refrigerator.

Cells were plated by placing a droplet containing 100–300 cells into the Cell-Tak coated well of the dish at a density of 10^5^cell/ml in a 1–3 μl volume droplet. Once placed into the cell dish the well was covered with a 22 mm by 22 mm autoclaved glass coverslip (22 × 22 mm Fisherfinest Premium Cover Glasses, Fisher Scientific) and the cell dish was placed into the incubator for half an hour. Upon removal from the incubator, the coverslip was taken off and 5 ml of DMEM was added to the cell dish, before returning it to the incubator at 37 °C and 5% CO_2_. The dish was left in the incubator for a minimum of 3 h before being placed onto the microbeam endstation for observation.

To observe the cells using the RARAF endstation, cells were stained using Hoechst 33342 (Thermo Fisher Scientific, Waltham, MA) at a concentration of 100 nM in DMEM [18]. The cells were incubated at 37 °C and 5% CO_2_ with the stain for 30 min and then the medium was replaced with fresh DMEM supplemented with 10% Foetal Bovine Serum and 1% Pen/Strep prior to observation.

### 2.12. Microscopy

A detailed description of the microscope system used on Microbeam II, including details on the hardware and software systems used to control the endstation and camera can be found in Bigelow et al. [19]. It consists of a modified Nikon Eclipse 600FN (Nikon, Tokyo Japan) with a Mad City Labs LP-200 low-profile nano-positioner stage (Madison, WI). The system remains unchanged from the system explained by Bigelow et al. with the exception of a new SOLA light engine(Lumencor, OR) light source has been added, this light source was used in conjunction with a Semrock DA/FI/TX-B Triple-band Filter set (Semrock, NY). The system does not use a focus compensation system, as a result, the focus has to be manually readjusted every few hours. The images see in Fig. 4 were stored as TIFs, in a 512 × 512 pixel image with a 0.8 μm × 0.8 μm pixel size using a 200 ms exposure time. Outside of the time images were taken the cells were not illuminated.

## 3. Results

### 3.1. AMOEBA experiment stability

**Fig. 3:**
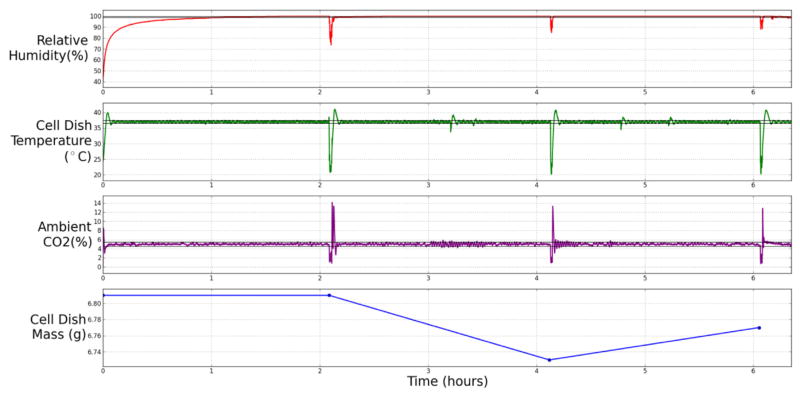
A 6-h AMOEBA evaporation experiment showing continuous monitoring of relative humidity, cell medium temperature, CO_2_ levels within the chamber and cell dish mass. The mean and RMS error values for the data can be seen in Table 1. The black lines on the first three plots show the acceptable boundaries of the signal.

Fig. 3 shows the stability of the environmental conditions in the AMOEBA. A cell dish (4.71 g) was filled with 2 ml of DMEM for a total weight of 6.81 g. Over a 6 h-long observation period the cell dish mass dropped to 6.77 g, this means that 0.03 ml of cell medium was lost over 6 h. The desired environmental conditions were: cell media temperature 37 °C ±2 °C, ambient CO_2_ 5% ± 0.5% and relative humidity 99.5% ± 0.5%. The spikes seen every two hours in the relative humidity, cell dish temperature and ambient CO_2_ are caused by the removal of the cell dish from the system so that it can be weighed and would not occur if the system was left for normal operation.

When first inserted into the AMOEBA the cell dish temperature reached 37 °C within 6 min, reaching a maximal overshoot temperature of 40 °C, with the dish temperature above 38 °C for 121 s. The same occurred when the dish was replaced after being weighed. The dish temperature spiked to a maximum of 41 °C, these temperatures returned back to the acceptable limits within 130 s. The mean dish temperature over the 6 h period was 36.840 °C and the RMS Error was 2.754 °C including the spikes experienced when the dish was removed the temperature oscillated within the acceptable boundaries at a frequency of 10.2 mHz. The CO_2_ within the chamber initially peaked at 8%, settling to 5% within 145 s after inserting the dish. When the dish was removed from the chamber to weigh it, ambient CO_2_ spiked to a maximum of 14.2% after the chamber was closed. The mean settling time of the system after being disturbed was 371 s (6.19 min), the signal oscillated within the acceptable boundaries at an approximate frequency of 8mHz. Over the 6 h period, the mean CO_2_ value was 4.994% and the RMS Error of the CO_2_ signal was 0.370%, including the spikes caused by removing the cell dish. In the times between the spikes, the means and RMS error values can be seen in Table 1.

During the undisturbed periods of operation when the system exceeded the bounds the AMOEBA successfully restored the parameter to the acceptable limits within a maximum of 1.2 min for cell dish temperature and 36 s for ambient CO_2_. Once the environment was established during the 6 h period, the relative humidity did not drop below 99% other than the times the system was disturbed to measure the mass of the cell dish.

Two other similar experiments were carried out using the AMOEBA; the results of these experiments can be seen in Table 2. In all three experiments the maximum mass gain was 0.108 g h^−1^ and the maximum mass loss was 0.067 g h^−1^. The mean mass loss was 6 mg h^−1^ with a variance of 0.002 g h^−1^. The control experiments shown in Fig. S1, record cell medium evaporation over 1.5 h periods outside of the environmental chamber with no cell medium heating show a mean evaporation of 0.567 g h^−1^ with a variance of 0.0002 g h^−1^. As a result the AMOEBA system has improved the loss of medium due to evaporation by a factor of 94.5 times. If the mean evaporation rate was sustained over a 12 h period the dish would lose 72 mg and over a 36 h period the dish would lose 216 mg.

### 3.2. Cell culture results

**Fig. 4:**
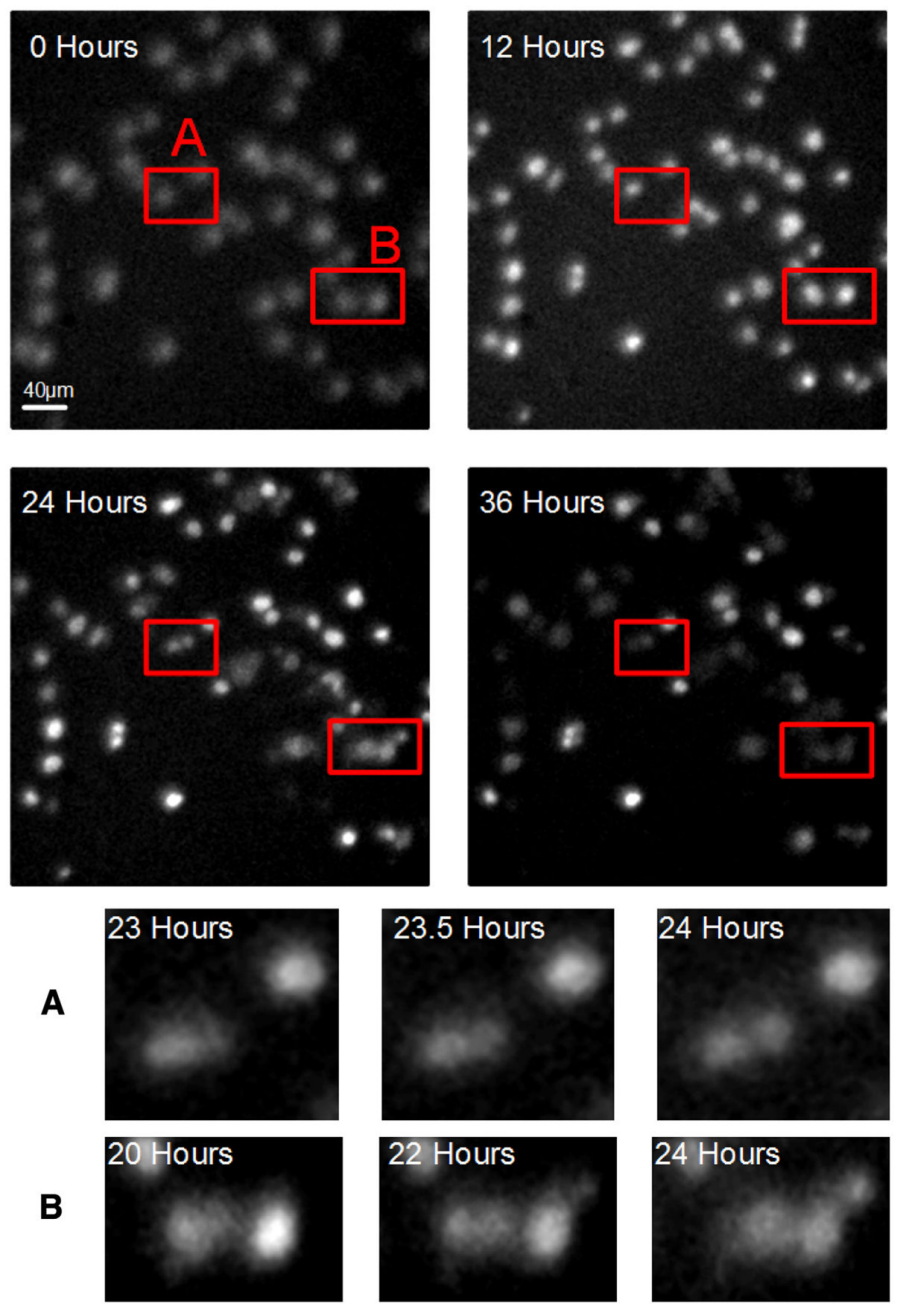
This figure shows cells in the AMOEBA device. The cells were stained with Hoechst 33342 at 100 nM and were viewed through a 20x objective. The red box shows locations where the cells divide over the 36-h observation. These red boxes are expanded in the bottom half of the figure. The 0 h image also shows a 40 μm scale bar that is also applicable to the 12, 24 and 36 h images. (For interpretation of the references to colour in this figure legend, the reader is referred to the web version of this article.)

A dish of Hoechst 33342 stained HeLa cells was examined under the microscope for 36 h; during the 36-h experiment, the AMOEBA regulated the environment at incubator conditions and an image was taken every 10 min. Cell division was successfully observed with two examples are highlighted in Fig. 4. A time resolved nucleus count is also provided in Fig. S2.

## 4. Discussion

### 4.1. The design of the AMOEBA system

The AMOEBA was designed to be a modular environmental control system that would be flexible enough to be applied to many situations beyond the area of environmental control. The single bus design allows modules to be easily added to and removed from the AMOEBA system. Its flexibility is also attested to by two facts: firstly that the AMOEBA system has worked on two beamlines, the Permanent Magnet Microbeam and Microbeam 2, at the RARAF facility, secondly, the AMOEBA system is in use in two VMB laboratories each with their own unique environmental setups(RARAF at Columbia University and at the Surrey Ion Beam Centre at Surrey University) [20].

The AMOEBA system is portable and can be moved between systems with little effort. The AMOEBA system can be adapted to almost any microscope since it would only require a new environmental shroud rather than require the whole system to be redesigned. If another parameter needs to be controlled, such as oxygen, the sensor would simply require a new module to be developed before adding it to the system.

Another testimony of the power and flexibility of the AMOEBA system is its ability to use standard communication protocols, this is shown by the RS-232 module that is able to interface with a Jenco 6230N (Jenco Quality Instruments, San Diego, CA). This feature of the AMOEBA system means that items of lab equipment with standard interface protocols such as USB, RS232 or GPIB, can be used as part of the AMOEBA control system.

### 4.2. Environmental stability of the RARAF AMOEBA environment

In this work, we demonstrated that the AMOEBA is capable of creating an incubator-like environment for extended live cell experiments. During the 6 h test, the temperature of the cell media stayed within the desired temperature bounds (37 °C ±1 °C) once it had reached its desired temperature. The four occasions when the temperature did exceed these bounds the temperature of the cell dish returned to desired range within 2 min. Likewise, the CO_2_ levels stayed within the desired bounds of 5% ± 0.5% over the whole 6 h period; when it did briefly stray from outside this range it returned within 2 min.

When starting the system the temperature and the CO_2_ values briefly overshot their desired set point, however, these parameters settled within minutes. Of these two values the temperature is the most critical for cell health, but the temperature spike is too short to damage the cells [21]. This overshoot could also be reduced by improved tuning of the system. The relative humidity took over an hour to stabilise but this is not an issue because evaporation is a slow process, the relative humidity would stabilise faster if the volume of the environmental shroud is reduced or the airflow rate is increased.

When the cell dish was removed, the CO_2_ initially dropped to below 1% due to the chamber being opened, the relative humidity also dropped for the same reason but because there was still a net flow of humid air and CO_2_ into the chamber, and the chamber was not opened for long enough for more CO_2_ or humid air to diffuse out of the chamber, as a result the relative humidity and CO_2_ did not drop to ambient levels. The temperature measured by the thermocouple measuring cell medium temperature dropped when the cell dish was being weighed because when the cell dish was removed to be weighed the dish and the thermocouple measured the temperature of the air in the chamber. When the dish was replaced the CO_2_ experienced a spike because the variable valve had opened more to compensate for the drop in CO_2_, resulting in an increased flow of CO_2_ in the shroud causing a spike when the chamber was closed.

### 4.3. Cell culture

We used the AMOEBA to observe cells for a 36 h period including instances of cell division. The unique culture conditions required to irradiate cells VMB, likely reduced and delayed cell division during this time. This delay is likely to have been caused by the polypropylene substrate, this can be deduced because Hoechst 33342 has previously been demonstrated to not be toxic to HeLa cells [22], and the AMOEBA system also regulated the environment at incubator conditions, this leaves the polypropylene substrate as the likely reason for the reduced cell division. It is also true that more cell division took place than was noticed since most of the cell division occurred after 24 h when the Hoechst had faded making the cells difficult to observe. Regardless of the delay, this shows that the AMOEBA system is capable of creating an environment in which cells are able to reproduce. It is likely that we would have observed more cell division had we left the experiment to run for a longer period of time. This experiment was stopped at 36 h because the stain had started to fade. The use of an alternative stain with a longer lifetime, or a bright field microscope without the use of stains, could increase observation time indefinitely [23]. The fact that all the observed cell division occurred in the last 12 h of the experiment also demonstrates that the AMOEBA system maintained a level of osmolality that allowed cell division to occur throughout the whole time period.

## 5. Conclusions

The AMOEBA system provides a flexible low-cost alternative to commercial environmental control systems. Unlike commercial systems, its modular design means it can be rearranged and within minutes be ready for almost any task that is required of it. The AMOEBA system opens up the potential of VMBs to irradiate and observe cells for extended periods of time. This system will enable microbeams to carry out experiments that were not possible previously, paving the way for microbeams to be able to assess cell motility, explore the effects of radiation over several cell divisions and to observe mutagenic effects of particle radiation without having to remove the cells from the microbeam.

## Supplementary Material

Supplemental

## Figures and Tables

**Table 1:** Mean and RMS Error Values for the AMOEBA experiment shown in Fig. 3. The number at the top of each column of Mean and RMS Error correspond to the 2-h section between each of the mass measurements.

	0–2.09 h		2.19–4.13 h		4.23–6.06 h		With Spikes	
			
	Mean	RMS Error	Mean	RMS Error	Mean	RMS Error	Mean	RMS Error
Relative Humidity (%)	96.469	6.560	99.925	0.151	99.990	0.002	98.668	23.947
Cell Medium Temperature (°C)	36.918	0.978	36.979	0.342	36.977	0.314	36.840	2.754
CO_2_ (%)	4.979	0.271	5.000	0.231	4.997	0.188	4.994	0.370

**Table 2:** Mass for three AMOEBA experiments. During the 6 h experiments the cell dish was weighed approximately every 2 h. The maximum gain in cell dish mass was 0.12 g h^−1^ and the maximum cell media evaporation was 0.06 g h^−1^.

Experiment	Mass (g)				

	0 h	2.04 h	3.97 h	5.85 h	Total Mass Gain
1	6.65	6.87	6.95	6.96	0.31
	0 h	2.09 h	4.12 h	6.05 h	
2 (shown in Fig. 2)	6.81	6.81	6.73	6.77	−0.04
	0 h	2.12 h	4.15 h	6.10 h	
3	6.6	6.51	6.57	6.44	−0.16

## Appendix A. Supplementary data

Supplementary data associated with this article can be found, in the online version, at http://dx.doi.org/10.1016/j.snb.2016.08.076.
