# Supplementary material for: Automated microbeam observation environment for biological analysis—Custom portable environmental control applied to a vertical microbeam system
Source: Sens Actuators B Chem. Author manuscript; Available in PMC 2018 Mar 5. (PMC5836785; doi:10.1016/j.snb.2016.08.076)
Supplement: Supplemental [file NIHMS932377-supplement-Supplemental.docx]

Supplementary information for: Automated Microbeam Observation Environment for Biological Analysis – Environmental Control, Vertical Microbeams Beams and Time-lapse Microscopy

Matthew J. England^1^, Alan W. Bigelow^2^, Michael J. Merchant^3^, Eirini Velliou^4^, David Welch^2^, David J. Brenner^2^, Karen J. Kirkby^5^

Figure S1: A) The effect of the temperature of the air entering the AMOEBA environmental shroud on cell dish mass, the experiments were carried out with an air flow of 2 scfh. B) The effects of air flow entering the humidifier on cell dish mass, the temperature of the air entering the chamber was 35°C. The experiments were carried out using DMEM the cell medium being heated to 37°C using the under dish heater. The control experiments were carried out without the environmental shroud and without dish heating to replicate the condition of a standard microbeam experiment without the AMOEBA system. Each experiment takes 1.5 hours with the cell dish being weighed every 30 minutes. Together these figures show the AMOEBA systems ability to prevent the evaporation of cell medium.


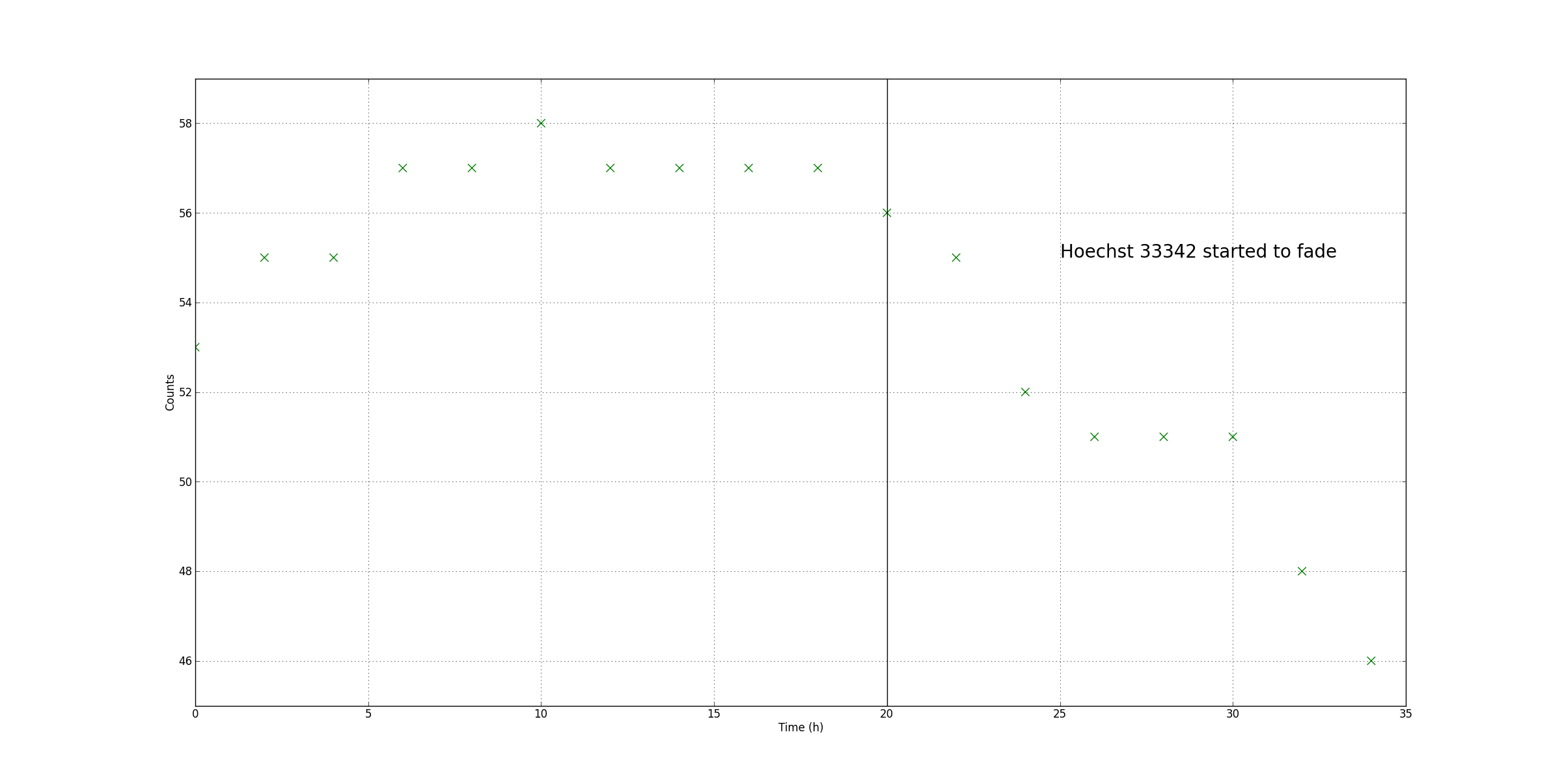


Figure S2: The above graph shows the number of cells visible at 2 hour intervals of the AMOEBA experiment shown in Figure 4. The cells were counted by hand using the Cell Counter plug in for Image J. After 20 hours the Hoechst 33342 started to fade, in many cases this made the cells difficult to count and in some cases the cells disappeared completely.
